# Supplementary material for: A Study of Phytochemistry, Genoprotective Activity, and Antitumor Effects of Extracts of the Selected Lamiaceae Species
Source: Plants (Basel). 2021 Oct 27;10(11):2306. doi: 10.3390/plants10112306 (PMC8623784; doi:10.3390/plants10112306)
Supplement: Supplementary file 1 [file plants-10-02306-s001.zip › plants-1426783-supplementary.pdf]

plants

MDPI

Table S1. The content of polyphenolic components (total phenolic (TPC), phenolic acid (PAC), total flavonoid (TFC) and flavonol content (FC)) of the selected plant extracts presented on three concentrations (100, 250, and 500 μg/mL) as means ± standard deviation.

**Species**	**Extract**	**TPC (mg GAE/g)**			**PAC (mg CAE/g)**			**TFC (mg QE/g)**			**FC (mg QE/g)**		
**concentration (μg/mL)**		**100**	**250**	**500**	**100**	**250**	**500**	**100**	**250**	**500**	**100**	**250**	**500**
Mo	**Methanolic**	32.40±1.22	79.40±3.78	147.77±5.74	19.37±2.31	58.26±8.34	176.04±6.42	4.17±0.25	11.25±1.13	21.16±2.05	nd	0.90±0.29	4.58±0.23
	**Ethanolic**	30.64±0.59	70.30±1.52	132.15±1.43	39.74±1.70	67.89±1.92	163.81±6.79	3.48±0.46	10.13±0.43	16.03±2.48	0.17±0.09	0.96±0.23	3.46±0.12
	**Aqueous**	31.72±0.34	74.22±1.39	145.85±3.74	13.81±1.28	40.48±2.31	132.70±5.59	0.87±0.28	5.97±0.22	15.19±0.84	nd	1.30±0.55	3.88±0.10
Mp	**Methanolic**	33.74±1.58	77.12±4.11	144.15±2.66	8.26±2.57	51.59±2.80	123.07±4.21	12.06±0.27	32.00±0.44	57.56±2.83	3.71±0.42	11.09±0.20	21.90±0.35
	**Ethanolic**	31.03±2.74	70.79±2.10	130.62±0.30	11.22±2.94	54.56±2.94	161.96±2.31	11.28±0.59	30.85±0.35	54.80±1.60	4.23±0.15	11.34±0.26	23.75±0.52
	**Aqueous**	33.15±0.80	76.27±2.64	146.01±2.97	2.33±1.11	16.41±1.70	86.41±1.70	4.32±0.11	21.31±0.34	43.02±0.83	1.26±0.15	5.33±0.21	10.07±0.26
Ob	**Methanolic**	7.68±0.37	19.71±0.40	38.76±2.63	nd	12.70±7.40	18.26±5.48	3.02±0.19	8.58±0.35	18.92±3.43	nd	0.96±0.47	3.58±0.12
	**Ethanolic**	11.59±0.60	29.14±0.37	55.14±1.52	11.96±0.64	19.00±3.33	10.85±5.25	3.23±0.19	10.28±0.33	18.67±0.78	0.36±0.12	2.67±0.21	5.98±0.07
	**Aqueous**	9.80±0.26	24.28±0.17	50.50±1.02	nd	nd	15.30±2.80	2.46±0.71	8.76±0.57	16.84±0.95	0.20±0.19	0.55±0.54	2.15±0.36
Ro	**Methanolic**	23.59±0.10	57.32±0.54	111.53±1.89	28.26±2.80	68.63±8.34	119.37±7.56	7.18±0.28	18.52±1.15	40.76±1.90	1.57±0.58	3.75±0.13	9.36±0.55
	**Ethanolic**	21.73±0.29	54.09±2.33	109.06±2.55	17.52±3.90	33.07±1.70	140.85±5.25	4.17±0.09	12.09±0.28	23.70±1.89	1.32±0.20	2.50±0.06	5.81±0.29
	**Aqueous**	29.82±2.48	76.37±0.74	139.42±2.19	23.44±1.11	33.44±4.44	116.78±6.67	6.03±0.41	14.82±0.38	29.05±0.34	2.84±0.23	5.77±0.43	12.02±0.68
So	**Methanolic**	29.27±0.85	70.79±2.16	131.89±0.65	18.26±0.64	46.78±6.19	103.44±2.94	9.48±0.32	26.93±0.67	53.09±1.86	4.17±0.36	8.84±0.39	14.87±0.72
	**Ethanolic**	27.87±0.99	69.26±5.77	134.89±2.91	11.59±1.70	50.11±10.94	99.00±8.39	7.09±0.30	19.95±0.05	43.86±1.43	2.54±0.20	6.91±0.20	13.83±0.10
	**Aqueous**	16.68±0.89	44.63±0.64	84.98±1.87	nd	30.85±2.31	64.56±5.56	8.86±0.91	20.50±0.14	39.98±0.85	3.09±0.18	8.01±0.30	14.73±0.07
Sm	**Methanolic**	21.99±0.41	55.17±1.87	106.67±3.64	19.74±2.31	64.56±3.33	130.11±1.11	3.85±0.19	10.47±0.53	26.16±1.03	2.48±0.35	4.39±0.27	9.09±0.65
	**Ethanolic**	28.49±0.78	66.52±1.21	134.56±0.49	5.67±1.11	44.93±3.57	105.67±9.09	4.04±0.23	12.30±0.22	28.92±0.57	1.40±0.12	4.06±0.31	8.35±0.55
	**Aqueous**	23.95±0.98	63.49±1.57	118.35±2.40	1.59±0.64	35.30±7.23	75.67±6.76	4.57±0.22	12.46±0.24	27.34±0.14	3.48±0.35	6.62±0.29	12.82±0.70

nd – not detected
